# Society Meetings

**Published:** 1918-07

**Authors:** 


### SOCIETY MEETINGS

**Brief reports and announcements of meetings of societies of Western New York. and those of general scope, are requested from Secretaries. Copy should be on hand the fifteenth of the month. Full transactions will be published at cost of composition.**

The **Buffalo Academy of Medicine** has elected the following officers for 1918-1819: President, Dr. F. Park Lewis; Secretary, Dr. James B. Cross; Treasurer, Dr. Bernard Schreiner.

**Buffalo Academy of Medicine**; Meeting May 22nd. Program: Water Purification and Disease by James M. Caird, A. S. C. E., Troy.

**Elmira Academy of Medicine** and The Chemung Co. Medical Society held a joint meeting on June 5th. Dr. W. A. Dewitt of Blossburg, Pa., gave a paper on "Fee Schedule to be Discussed."

The Medical Department of the **University of Buffalo** held its 43rd Annual Meeting, June 5, 6 and 7th at the College. Papers were read by the following physicians: "The Alkaloidal Derivatives of Ipecac in Therapeutics" by Dr. Edward W. Koch, Buffalo; "Pneumonia and Pneumococcic Differentiation" by Dr. A. M. Zillig, Buffalo; "Demonstration of the Colloidal Gold Reaction" by Dr. A. E. Zielinski, Buffalo; "The Medical Department of the U. S. Army in Relation to the War" by Dr. Thos. B. Woodson, Buffalo; "The Clinical Results of Tissue Transplantation" by Dr. Allen B. Kanavel, Chicago, Ill.; "X-ray Diagnosis of Diseases of the Heart and Aorta" by Dr. I. H. Levy, Syracuse; "The Internal Ear and Brain Localization from the Viewpoint of the General Practitioner" by Dr. Lewis Fisher, Philadelphia; "The Influence of the War upon the Public Health Problem" by Dr. Chas. Hastings, Toronto; "The Newer Aspects of the Pneumonia Problem" by Dr. Alexander McPhedran, Toronto; and "Psycho Neuroses and the War with Clinical Demonstration" by Dr. Smith E. Jelliffe, New York City.

### Our Friends the Feebleminded.

The recent meeting here at Buffalo, of the American Association for the Study of the Feebleminded—May 31st and June 1st—had an importance quite out of proportion to the size of the association itself or the degree of attention which it attracted from the medical profession. Of the more technical aspects of the program this review will perforce have little to say, especially as the variety of that program and its new orientations were of more significance than even the ablest paper presented by the brilliant group of experts who were in attendance. The association was originally for the Care and Study of the Feebleminded, and its members predominantly superintendents of institutions for mental defectives or the insane. The problem as seen was a static one; the custodial idea was in control. But on the program of the meeting just concluded it had scant recognition; instead, extra-institutional care in various phases, industrial training and the relation of the schools to backward and degenerate children were to the fore, as well as papers dealing with the causes and prevention of feeblemindedness. We have come to see that the question is dynamic to a high degree, with far-reaching ramifications. The relation of mental defect to poverty and to crime is so well established, its by-products so menacing, and the question of what to do about it so urgent.

A year and a half ago Erie County had filled its quota of places in public institutions for such cases, and something more, had sixty-four inmates in the Brunswick Home on Long Island, at about double the per capita cost in state institutions, and a waiting list of at least sixty-five. This list contained merely certain of the more marked cases, in which our county agents had been able to overcome legal difficulties and family objections, and in no degree measured the strength of our reserves in citizens of that quality. What their numbers are we do not know today, but in the spring of 1917 a letter was sent from the office of the Superintendent of the Poor to each school in the county outside of the city of Buffalo, asking whether there were defective children in that school. The inquiry revealed about 585 such children. An accepted estimate of the proportion of defectives to the population is two per cent.

As a first step toward meeting this situation, last spring a colony for feebleminded boys was started at Akron by Dr. Bernstein of the Custodial Asylum at Rome. A building which had had a brief life as a sanitarium was rented with some sixty acres of ground, and a Roman cohort, under the charge of an excellent Scotch couple, took possession. "We move in," said Dr. Bernstein, "as the United States army does," and all honor is due to the woman who led that advance guard, and under whose hand that building, with the accumulated dust and dilapidation of seven years of disuse, grew into pleasantness and comfort. From the farmer's point of view it was a late beginning, and the season was bad. But the twenty boys there last summer nevertheless raised the winter vegetables needed by the colony and had "the best field of cabbage in Erie County." A number of the boys also found employment in the town, taking care of lawns and gardens, and with neighboring farmers. They have earned, up to date, $2,059.00. Ninety additional acres have now been rented, to be devoted to truck farming, and a second home has been opened, bringing the available accommodations up to forty. This second house is also a school, where the boys have daily lessons in such branches as they can master. Plans are also under way for one or two colonies of women in other towns of the county. Those women will do laundry work and cleaning, and a few of them can sew acceptably. No one will question that their labor will be in demand. These colonies, whose ultimate success must be held subjudice, accomplish certain definite things. If the feebleminded—like the rest of us—are to be kept out of mischief, they must be kept busy. In time, an institution comes to have a greater supply of certain kinds of labor than it can utilize; if some of these workers swarm successfully from the mother hive, place is made for just so many on the waiting list who sorely need institutional care. The community gains labor to which it is entitled, and at least a measure of the support of these dependents is lifted from its shoulders. "Why lock up that labor," asks Dr. Bernstein pertinently, "and then get in a lot more just like it through Ellis Island to fill the gap?" Moreover, the defective himself is brought by so much nearer to normal social relations. Such colonies, as a step toward "The Rehabilitation of the Mentally Defective—"Dr. Bernstein's paper at the recent meeting—are laboratories in which a human product is being tested, and their experience should be studied with sympathetic attention and the open mind.

Those who attended these meetings will remember the statements of Dr. H. H. Goddard that no normal child should be institutionalized and every abnormal one should be, and that the state of mind in which the normal person faces work requiring trained skill is the condition of the defective in regard to the simplest job. But each, in his own measure, can be taught, and it may prove that a considerable proportion of the mentally defective, after the institution has trained them in some routine occupation and developed in them certain habits of conduct, can be safely returned to a measure of community life. It may even prove that certain of the high-grade defectives need less than that degree of institutionalizing. It was with this question in mind that the Vassar Club arranged a preliminary meeting, on the evening of May 30, for the discussion of the industrial training and after-control which would best fit mental defectives for extra-institutional life, hoping to focus the discussion especially on our local conditions. The club, which published, a year ago, a brief report on mental defectives here at Buffalo, is attempting an investigation of the records of pupils in our special classes for defectives who remained until the age of sixteen and were then set free, after the custom of the day, without a trade and without supervision, to go where they would. If this inquiry reveals, as the first data seems to indicate, that even under these conditions a fair proportion show industrial capacity, the duty of the community to such boys and girls is pretty clear. In his paper, Dr. George E. Smith, of our Dept. of Education, dealt with this subject in its wider phases—the just claims upon us, for training suited to their needs, of the mental defective and his near-kin, the backward child. The possibilities latent in the latter class are one of the encouraging features of the situation. In a recent survey of the school population in the Locust Point district of Baltimore—a slum district—"the subnormal children were grouped according to their prospective adult efficiency into three groups, those who could never be independent, those of almost certain partial dependency, those of merely possible dependency," and " a consecutive series of fifty cases of backward children between the ages of fourteen and sixteen years, who applied for a special permit to work, was studied for two years; these children had not passed the fifth grade, otherwise they would have been entitled to a permit. The average wage of these children at the end of two years was $6 a week; this no doubt may have been due to unusually favorable labor conditions."

"As to the mental level of the children, thirteen had a mental age of nine years when they applied for a permit in 1914, i.e., they were at least "five years retarded." But two of these children, then "five years retarded," are at present attending night school; one of them is making $7 a week as a factory worker, the other is making $8 a week as a huckster."

Such facts as these, together with the established teachableness, within certain limits, of the moron of good temperamental qualities, indicate quite definitely the directions in which we should advance our treatment of the laggard child. Nowhere are the rules of thumb less to be trusted than in estimating the intelligence of such a child; "they are apt to leave us in the lurch when we most require them, when we would like to have some means of determining whether the individual child will be able later to become an independent, self-supporting worker.—What "mental level" must be attained if the individual is to be self-supporting?—Is it the same for urban and country life, for North and South?*asks Dr. Macfie Campbell, from whom we have been quoting. Is it indeed true that the man whom Massachusetts rejects as feebleminded may become a leading citizen in Oklahoma?

*Mental Hygiene, April, 1918, pg. 235.

One of the outstanding features of the meeting was the marked differences of opinion which were elicited. This means a new field, the laboratory spirit, and the challenge of experts at every step in unravelling its problems, which go deep into the fabric of our social organization and government.

In solving those problems Buffalo can do its full share. We have able leaders, a devoted body of trained workers, and constantly widening public support. Nor do we lack material. How many of us there are is still a matter of surmise; but the rumors from army examining boards are not flattering, and it is seriously proposed to recognize a new mental grade, between the moron and the normal man—the "simples"—in confident expectation that an ample proportion of one's fellow-citizens will qualify under it. But the question of numbers will soon be settled. The Sage bill, passed this year, established a State Commission on the Feebleminded, which provides, among other measures, for a census of the feebleminded in the State, under a sweeping definition of mental deficiency—"any person afflicted with mental defectiveness from birth or from an early age, to such an extent that he is incapable of managing himself and his affairs, or of being taught to do so, who for his own welfare and the welfare of others, or for the welfare of the community, requires supervision, control or care, and who is not insane or of unsound mind to such an extent as to require his commitment to an institution for the insane as provided by the insanity law." The commission is to consist of three members, a physician, the fiscal supervisor of state charities, and the secretary of the State Board of Charities. Among other duties, they are to prepare a general commitment law, and provide clinics, colonies and necessary institutional accommodations. All feebleminded persons in the state are to be recorded. The Commission may commandeer the services of any state agency, the Dept. of Education being specifically mentioned.

It is a great step forward, but if the problems of that wide and tangled field are to be cleared up, there must be the full co-operation of the medical profession as well.

—ELIZABETH M. HOWE.

---

**The American Academy of Ophthalmology and Oto-**

**Laryngology** will hold its 23d annual meeting at Denver, Aug. 5-7. Dr. Lee Masten Francis of Buffalo is the Secretary.

---

The regular meeting of the **Medical Society of the County of Erie** was held on Monday, June 17th, at 9 p. m., in the Buffalo Medical College.

In the absence of President Cott, 1st Vice-President James E. King presided.

The Secretary read the minutes of the regular meeting held April 15th, also the meeting of the Council held May 13th, and meeting of the Council held June 17th, which were approved as read.

Dr. Jacobs, Chairman of the Committee on Membership presented the names of Dr. George W. Schaefer, 967 Jefferson Street, for re-election to the Society. Dr. Schaefer having resigned at the time he left Buffalo, and also Dr. Guy L. McCutcheon, 520 Potomac Avenue, Buffalo, for re-instatement. The Membership Committee recommended their re-election and re-instatement.

On motion the Secretary was instructed to cast the ballot of the Society for the re-election and re-instatement of these men.

Reports of committees being called for Dr. Woodruff presented a verbal report of part of the work, done by the Delegates to the State Society. He also reported briefly for the Committee, relative to Medical Fees.

Dr. Lytle made a brief verbal report for the Committee on the Home for Feebleminded at Akron, all of which were accepted.

Dr. Lytle stated that under the by-laws the Treasurer is required to report the names of all those who are in arrears with their dues at this meeting. On motion the reading of these names was dispensed with.

The Secretary called attention to the resolution adopted by the Council, relative to the payment of State and County dues of members who are in the Federal service, and also called attention to the action taken by the State Society, regarding this question.

On motion of Dr. Gram it was resolved that the question of remission of State and County dues of all such members who are in arrears on December 31st, 1918, by reason of the fact that they are in the Federal service be left to the Council with power. The motion was adopted.

Dr. Lytle, President of the 8th District Branch, stated that although the last meeting of this Branch was held in Buffalo in 1917, the members were inclined to return to Buffalo for their 1918 meeting, on account of the better facilities offered both for reaching the city and for holding meetings.

On motion of Dr. Gram the 8th District Branch was invited to hold its next meeting in Buffalo in September.

Moved by Dr. H. H. Glosser that it is the sense of this meeting that physicians of Buffalo should live up to the State Law and City Ordinance and report all Venereal Diseases coming to their notice. The motion was adopted.

The paper of the evening was then presented by Dr. Walter S. Goodale, Superintendent of Hospitals and Dispensaries on the subject of "The Control of the Venereal Infection."

This paper was discussed generally by members present and heartily recommended by every speaker.

The meeting then adjourned.

FRANKLIN C. GRAM, M. D.,
Secretary.

